# Lycopene Inhibits Activation of Epidermal Growth Factor Receptor and Expression of Cyclooxygenase-2 in Gastric Cancer Cells

**DOI:** 10.3390/nu11092113

**Published:** 2019-09-05

**Authors:** Hwana Han, Joo Weon Lim, Hyeyoung Kim

**Affiliations:** Department of Food and Nutrition, Brain Korea 21 PLUS Project, College of Human Ecology, Yonsei University, Seoul 03722, Korea (H.H.) (J.W.L.)

**Keywords:** apoptosis, cyclooxygenase-2, epidermal growth factor receptor, gastric cancer, lycopene

## Abstract

Reactive oxygen species (ROS) contribute to the oncogenic phenotype of cancer cells by acting as signaling molecules for inducing proliferation. ROS are known to activate the epidermal growth factor receptor (EGFR), which causes the activation of the Ras/mitogen-activated protein kinases (MAPKs) pathway. The Ras-dependent pathway promotes the activation of nuclear factor-kappa-light-chain-enhancer of activated B cells (NF-κB), a transcriptional modulator of cyclooxygenase-2 (COX-2) that induces cell proliferation. Lycopene is a potent antioxidant carotenoid and is responsible for the red color of fruits and vegetables. This study aims to investigate whether lycopene inhibits proliferation and induces apoptosis in gastric cancer AGS cells by suppressing the EGFR/Ras/MAPK and NF-κB-COX-2 signaling axis. Lycopene decreased cell viability and increased apoptotic indices (DNA fragmentation, apoptosis inducing factor, cleavage of caspase-3 and caspase-9, Bax/Bcl-2 ratio). Lycopene reduced the level of intracellular and mitochondrial ROS and decreased the activation of the ROS-mediated EGFR/Ras/extracellular signal-regulated kinase (ERK) and p38 MAPK pathways, thus leading to attenuation of the DNA-binding activity of NF-κB p50/p50 and the level of COX-2 gene expression. These results show that lycopene-induced apoptosis and inhibition of proliferation occur via inhibition of ROS-activated EGFR/Ras/ERK and p38 MAPK pathways and NF-κB-mediated COX-2 gene expression in AGS cells. In conclusion, consumption of lycopene-enriched foods could decrease the incidence of gastric cancer.

## 1. Introduction

Gastric cancer is the fourth most common cancer worldwide and is ranked second in mortality rate [1]. The most common histologic type is gastric adenocarcinoma, which accounts for over 90% of gastric tumors. Infection by *Helicobacter pylori* (*H. pylori*), high intake of salty foods, smoking, and alcohol consumption are known to be significant risk factors for gastric cancer [2,3,4]. These pathogenic factors are reported to be related to oxidative stress in gastric tissues.

Cancer cells are known to generate a higher amount of reactive oxygen species (ROS) compared to normal cells [5]. Excessive quantities of ROS result in tissue damage, stimulation of cell proliferation, DNA mutation, and resistance to chemotherapy, which contributes to cancer progression [6]. Protein nitration (3-nitrotyrosine), a biomarker for protein oxidation, and 8-oxo-7,8-dihydro-2-deoxyguanosine (8-OHdG), a byproduct of oxidative DNA damage, are reported to be markedly increased in gastric cancer tissues [7,8], suggesting that oxidative stress is closely linked to the pathological processes of gastric cancer. 

ROS participate in the initiation and progression of cancer by acting as a second messenger for oncogenic signaling pathways. One of the possible targets activated by ROS is the epidermal growth factor receptor (EGFR). Oxidative stress in rat gastric epithelial cells and human squamous carcinoma cells induces the phosphorylation of EGFR tyrosine residues [9,10]. Through activation of its downstream effectors, EGFR modulates cell migration, differentiation, adhesion and proliferation and thus, tumor cell survival [11]. The overexpression of EGFR is associated with a more advanced stage of the gastric cancer and its spread to lymph nodes, and consequently, with a poor prognosis [12,13,14]. Therefore, increased oxidative stress might facilitate the progression of gastric cancer via EGFR activation. 

Ras and mitogen-activated protein kinases (MAPKs) are the essential downstream kinases that mediate the pathophysiological responses of EGFR. Ras, a member of the superfamily of small GTPases, functions as a molecular switch by cycling between GTP-bound and GDP-bound states. Ras transduces signals from the cell membrane to the nucleus through the regulation of downstream kinases [15]. The activation of EGFR leads to its dimerization and to its tyrosine residue autophosphorylation, which in turn facilitates the change from the inactive GDP-bound Ras to the active GTP-bound Ras [16]. GTP-bound Ras contributes to the activation of the MAPK pathway, which regulates cell growth and cell proliferation [17,18]. Three well-characterized subfamilies of MAPKs that control a vast array of physiological processes are extracellular signal-regulated kinases (ERKs), jun kinases (JNKs) and p38 MAPK. ERKs function in the control of cell division, and ERK inhibitors are being explored as anticancer agents. JNKs and p38 MAPK have been related to inflammation and immunity [17]. 

MAPKs serve as upstream signals for the transcription factor, nuclear factor-kappa-light-chain-enhancer of activated B cells (NF-κB) [19,20]. Constitutive activation of NF-κB in gastric cancer cells has been reported [21,22]. The activation of NF-κB plays a crucial role in various aspects of cancer development because NF-κB regulates the transcription of genes responsible for cell survival and apoptosis. In particular, NF-κB promotes cell growth and suppresses apoptosis by regulating the gene encoding anti-apoptotic bcl-2 as well as proto-oncogenes such as c-myc and the cyclin D1 gene [23]. 

One of the essential target genes modulated by NF-κB encodes cyclooxygenase-2 (COX-2), a regulator of cell proliferation. The promoter of the human COX-2 gene is known to contain the binding site for NF-κB [24,25]. Aberrant upregulation of the COX-2 gene facilitates the development of cancer through angiogenesis and reduced apoptosis [26]. High levels of COX-2 and COX-2 mRNA in gastric tissues of gastric cancer patients have been reported [27,28]. In addition, COX-2 specific inhibitors have been shown to significantly induce apoptotic cell death and inhibit cell proliferation in an in vitro model of gastric cancer [29]. Taken together, these studies have demonstrated the involvement of the EGFR/Ras/MAPK signaling pathway in the activation of NF-κB, the induction of COX-2, and the proliferation of gastric cancer cells. Therefore, COX-2 appears to be a promising therapeutic target for the treatment of gastric cancer.

Lycopene belongs to the carotenoid family and contributes to red-orange color of fruits and vegetables including tomato, watermelon, guava, and pink grapefruit. Lycopene has strong singlet oxygen quenching capacity, which is two-fold and one hundred-fold greater than the capacity of β-carotene and α-tocopherol, respectively [30]. Epidemiological studies have demonstrated that high dietary intake of lycopene is inversely correlated with the risk of gastric cancer [31,32]. Lycopene inhibits oxidative damage to DNA and suppresses cell growth by downregulating redox-sensitive signaling pathways, including MAPK and NF-κB pathways in human prostate cancer, breast cancer, and hepatocellular carcinoma cell lines [33,34,35]. Previously, we showed that lycopene decreased the formation of 8-OHdG, and suppressed the ROS-activated Jak1/Stat3 and Wnt/β-catenin pathways in H. pylori-infected gastric epithelial cells [36,37]. These findings suggest that lycopene may suppresses cancer cell growth by reducing the level of ROS and thus, the impact of ROS on redox-sensitive signal transduction regulation of cell proliferation. We hypothesized that lycopene might suppress the oxidative stress-associated pathological responses associated with gastric cancer.

The present study was carried out to examine the effect of lycopene on proliferation and apoptosis and to determine the impact of lycopene on the levels of cellular ROS, activated EGFR and Ras, MAPKs and COX-2 in gastric cancer AGS cells. 

## 2. Materials and Methods 

### 2.1. Cell Line and Culture Condition

The human gastric cancer cell line AGS (gastric adenocarcinoma; ATCC CRL 1739) was obtained from the American Type Culture Collection (Rockville, MD, USA). Cell line identity was authenticated by standard short tandem-repeat-based DNA profiling (STR, Hendersonville, TN, USA). The cells were grown in a complete medium of RPMI 1640 (GIBCO, Grand Island, NE, USA) supplemented with 10% fetal bovine serum, 2 mM glutamine, 100 U/mL penicillin, and 100 μg/mL streptomycin (Sigma, St. Louis, MO, USA). The cells were incubated at 37 °C under a humidified atmosphere of 95% air and 5% CO2. Cultured cells were routinely tested for mycoplasma contamination by PCR amplification for mycoplasma pulmonis UAB CTIP, mycoplasma penetrans HF-2, and mycoplasma synoviae 53.

### 2.2. Lycopene Treatment

Lycopene (all-trans lycopene, L9879, Sigma,) was dissolved in tetrahydrofuran. The AGS cells were incubated with lycopene (0.5, 1, or 2 μM, Sigma) dissolved in tetrahydrofuran for 1 h (for measurement of intracellular ROS), for 24 h (for determination of DNA fragmentation and levels of apoptosis inducing factor (AIF), Bax, and Bcl-2), or for 24 and 48 h (for measurement of cell viability). In addition, in a second series of experiments the cells were incubated with 2 μM lycopene for 2 h (for immunoprecipitation of phosphorylated tyrosine and EGFR interaction), for 4 h (for determination of EGFR phosphorylation and Ras levels in membrane and whole-cell extracts, as well as the GST-fusion pull-down assay for Ras activation), or 24 h (for assessing MAPK activation, NF-κB-DNA binding activity, and the levels of NF-κB and COX-2). The control cells were treated with a vehicle tetrahydrofuran (THF) without lycopene and expressed as “none”. Since lycopene is highly hydrophobic, it is not easily dissolved in cell culture media. Therefore, we added THF to the cells under 0.05% (vol/vol) as a vehicle. 

### 2.3. Determination of Cell Viability

The AGS cells were seeded in a 24-well plate (1 × $10^{4}$ cells/well) and then cultured overnight. Cell viability was assessed by direct counting using a hemocytometer and the trypan blue exclusion test (0.2%, trypan blue; Sigma).

### 2.4. Assessment of DNA Fragmentation

DNA fragmentation was measured by quantification of cytoplasmic oligonucleosome-bound DNA fragments. The AGS cells (1 × 10^4^ cells/well) contained in a 24-well plate were first lysed and then centrifuged at 200× *g* for 10 min. The amount of nucleosome in the cell lysate was evaluated by using a sandwich ELISA assay (Cell Death Detection ELISAPLUS kit; Roche Diagnostics GmbH, Mannheim, Germany). The relative amount of nucleosome-bound DNA in the cell lysate was expressed as an enrichment factor determined from absorbance measurements of the samples determined at 405 nm. 

### 2.5. Annexin V/Propidium Iodide (PI)—Staining Assay

Apoptosis was measured by flow cytometry using Annexin V–fluorescein isothiocyanate (FITC)/PI staining. The AGS cells were treated with lycopene for 24 h. The cells were collected, washed with ice-cold PBS, and resuspended in 200 μL 1X binding buffer containing Annexin V (1:50 according to the manufacturer’s instructions) and 20 ng/sample of PI for 15 min at 37 °C in the dark. Then, the number of viable, apoptotic and necrotic cells was quantified by flow cytometry (Becton Dickinson, Franklin Lakes, NJ, USA) and analyzed by the CellQuest software. Cells were excited at 488 nm and the emissions of Annexin V at 525 nm and PI were collected through 610-nm band-pass filters. At least 10,000 cells were analyzed for each sample. Apoptosis rate (%) = (number of apoptotic cells)/(number of total cells observed) × 100.

### 2.6. Measurement of Intracellular and Mitochondrial ROS Levels

For the measurement of intracellular ROS, the cells were treated with 10 μg/mL of dichlorofluorescein diacetate (DCF-DA; Sigma-Aldrich, St. Louis, MO, USA) and incubated in 5% CO2/95% air at 37 °C for 30 min. DCF fluorescence was measured (excitation at 495 nm and emission at 535 nm) with a Victor5 multi-label counter (PerkinElmer Life and Analytical Sciences, Boston, MA, USA). For the measurement of mitochondrial ROS, the cells were treated with 10 μM MitoSOX red (Life technologies, Grand Island, NE, USA) and incubated in 5% CO2/95% air at 37 °C for 30 min. The MitoSOX fluorescence was measured (excitation at 514 nm and emission at 585 nm) using a Victor5 multi-label counter (PerkinElmer Life and Analytical Sciences, Boston, MA, USA). ROS levels were determined from the relative increases in fluorescence.

### 2.7. Preparation of Whole-Cell Extracts, Membrane Extracts, and Nuclear Extracts

The cells were first trypsinized and then pelleted by centrifugation at 5000× *g* for 5 min. The pellets were suspended with lysis buffer (10 mM Tris (pH 7.4), 15 mM NaCl, 1% Nonidet P-40 and protease inhibitor complex) and extracted by drawing the suspension through a 1 mL syringe with several rapid strokes. The resulting mixtures were placed on ice for 30 min and then centrifuged at 13,000× *g* for 15 min. The supernatants were used as whole-cell extracts. To prepare membrane extracts, the supernatants were further centrifuged at 100,000× *g* for 1 h at 4 °C. The pellets were resuspended in lysis buffer (50 mM HEPES, 150 mM NaCl, 1 mM EDTA, and 10% glycerol) and used as the membrane extracts. For the preparation of nuclear extracts, the cells were lysed in hypotonic buffer (10 mM HEPES, 1.5 mM MgCl2, 10 mM KCl, 1 mM DTT, 0.5 mM PMSF, 0.05% Nonidet P-40, and 0.1 mM EDTA), followed by centrifugation at 13,000× *g* for 10 min. The pellets were resuspended in nuclear extraction buffer (20 mM HEPES (pH 7.9), 420 mM NaCl, 0.1 mM EDTA, 1.5 mM MgCl2, 25% glycerol, 1 mM DTT, and 0.5 mM PMSF) on ice and then centrifuged. The supernatants were collected and used as nuclear extracts. Protein concentrations were measured by using the Bradford assay (Bio-Rad Laboratories, Hercules, CA, USA).

### 2.8. Western Blot Analysis 

The whole-cell extracts and membrane extracts (40–60 µg protein/lane) were separated by (8–12%) SDS polyacrylamide gel electrophoresis, and transferred onto nitrocellulose membranes (Amersham, Inc., Arlington Heights, IL, USA) by electroblotting. The membranes were incubated with 2% non-fat dry milk in TBS-T (Tris-buffered saline and 0.2% Tween 20) for 1 h at room temperature. The proteins were probed using antibodies for H-Ras (sc-520), EGFR (sc-31155), p-Tyr (sc-7020), p-EGFR (sc-12351), p-ERK (sc-7383), p-p38 (sc7974-R), NF-κB p65 (sc-7151), Bcl-2 (sc-492), Bax (sc-526), AIF (sc-13116), Na^+^/K^+^-ATPase (sc-21712), caspase-3 (sc-7148), casapse-9 (sc-81663), and actin (sc-47778) (all purchased from Santa Cruz Biotechnology, Dallas, TX, USA), p38 (#9212, Cell Signaling Technology), ERK (#9102, Cell Signaling Technology), JNK (#9252S, Cell Signaling Technology), p-JNK (#9251, Cell Signaling Technology) and NF-κB p50 (23659, Upstate Biotechnology) diluted in TBS-T solution containing 2% dry milk and incubated overnight at 4 °C. After washing with TBS-T, the primary antibodies were detected using horseradish peroxidase-conjugated secondary antibodies (anti-mouse, anti-rabbit, anti-goat), and visualized with the enhanced chemiluminescence (ECL) detection system (Santa Cruz Biotechnology). Actin was used as a loading control. The level of Bax was compared to that of Bcl-2 and expressed as the percentage density ratio. 

### 2.9. GST-Fusion Pull-Down Assay for Ras Activation 

For use in the pull-down assay, the glutathione S-transferase (GST) fusion protein containing the Ras binding domain (GST-RBD) of Raf1 was prepared as follows. The cDNA encoding the Ras binding domain (RBD) of Raf1 was amplified by PCR and then subcloned into the pGEX-4T-1 vector at the BamHI-XhoI sites (Amersham Biosciences, Piscataway, NJ, USA). The resulting plasmid was used to transform BL23 Escherichia coli cells. GST-RBD was purified from the cell lysate by extraction of the supernatant fraction with glutathione-agarose beads. The pull-down assay was carried out by incubating a mixture consisting of 485 μL of AGS cell extract, 10 μg of GST-RBD and 15 μL of glutathione-agarose beads at 4 °C for 45 min. The beads were separated from the mixture by centrifugation, washed three times with lysis buffer, and then resuspended in SDS-sample buffer for Western blot analysis. Anti-Ras antibody was used to measure the total Ras in the mixture whereas anti-H-Ras antibody (sc-520, Santa Cruz Biotechnology) was used to measure the activated Ras. 

### 2.10. Immunoprecipitation for Detection of Phosphorylated Tyrosine and EGFR Interaction

AGS cell extract was prepared and combined with anti-p-Tyr antibody (sc-7020, Santa Cruz Biotechnology) and protein G/A. The resultant mixture was immobilized on agarose in RIPA buffer (25 mM Tris–HCl (pH 7.5), 150 mM NaCl, 1% Triton X-100, 1 mM EDTA, 1 mM EGTA, 1 mM PMSF, 0.25% Nonidet P-40, and 0.5% sodium deoxycholate) and incubated overnight at 4 °C. The protein G-antibody-antigen conjugates were collected by washing the agarose three times with ice-cooled RIPA buffer. The final pellet was resuspended in 50 μL of SDS-sample buffer and boiled for 5 min. The preparation was subjected to Western blot analysis with anti-EGFR antibody (sc-31155, Santa Cruz Biotechnology). 

### 2.11. Electrophoretic Mobility Shift Assay (EMSA)

The shift assay was conducted with the NF-κB gel shift oligonucleotide probe (5′-AGTTGAGGGGACTTTCCCAGGGC-3′) obtained from Promega (Madison, WI, USA). The single-stranded oligonucleotides contained in the cellular extracts were end-labeled with [32P]-deoxyadenosine triphosphate (dATP) (Amersham Biosciences, Piscataway, NJ, USA) using T4 polynucleotide kinase (GIBCO). The radiolabeled oligonucleotides were separated from unincorporated [32P]-dATP by chromatography on a Bio-Rad purification column (Bio-Rad Laboratories) using Tris-EDTA buffer as eluant. Nuclear extracts were incubated with [32P]-radiolabeled probes in buffer (12% glycerol, 12 mM HEPES (pH 7.9), 1 mM EDTA, 1 mM DTT, 25 mM KCl, 5 mM MgCl2, 0.04 µg/mL poly[d(I-C)]) for 30 min at room temperature. For supershift analysis, antibodies directed against p50 or p65 were added to the nuclear extract 30 min prior to the reaction. The samples were analyzed by electrophoretic separation at 4 °C on a nondenaturing 5% acrylamide gel. After drying at 80 °C for 2 h, the gel was exposed to a radiography film on intensifying screens for 6 to 18 h at −80 °C.

### 2.12. Statistical Analysis

All values were expressed as mean ± S.E. of three independent experiments. For statistical analysis, one-way ANOVA, followed by Newman–Keul’s post hoc test, was used. A *p*-value of 0.05 or less was considered statistically significant.

## 3. Results

### 3.1. Lycopene Decreases Cell Viability and Induces Apoptosis in AGS Cells

**Figure 1:**
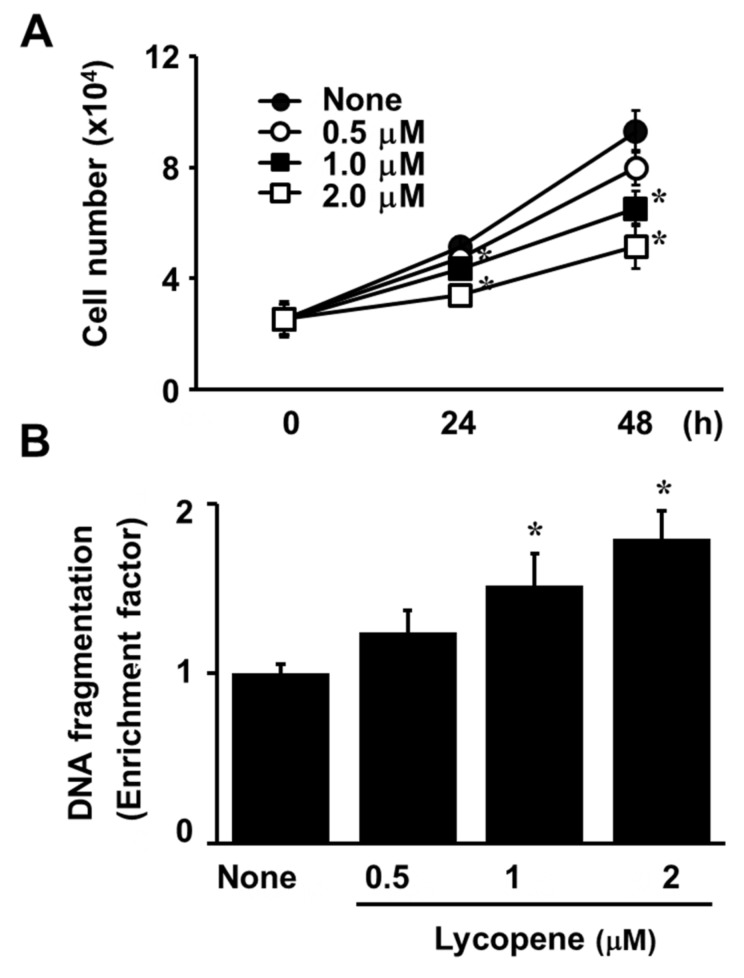
Lycopene inhibits cell proliferation and increases DNA fragmentation. (**A**) The cells were treated with the indicated concentrations of lycopene for 24 and 48 h. Cell viability was assessed using the trypan blue exclusion test. (**B**) The cells were treated with the indicated concentrations of lycopene for 24 h. DNA fragmentation was assessed by the amount of nucleosome-bound DNA in the cell lysates. The level of DNA fragmentation for untreated cells was set as 1. * *p* < 0.05 versus the cells treated with a vehicle tetrahydrofuran without lycopene (“none”).

**Figure 2:**
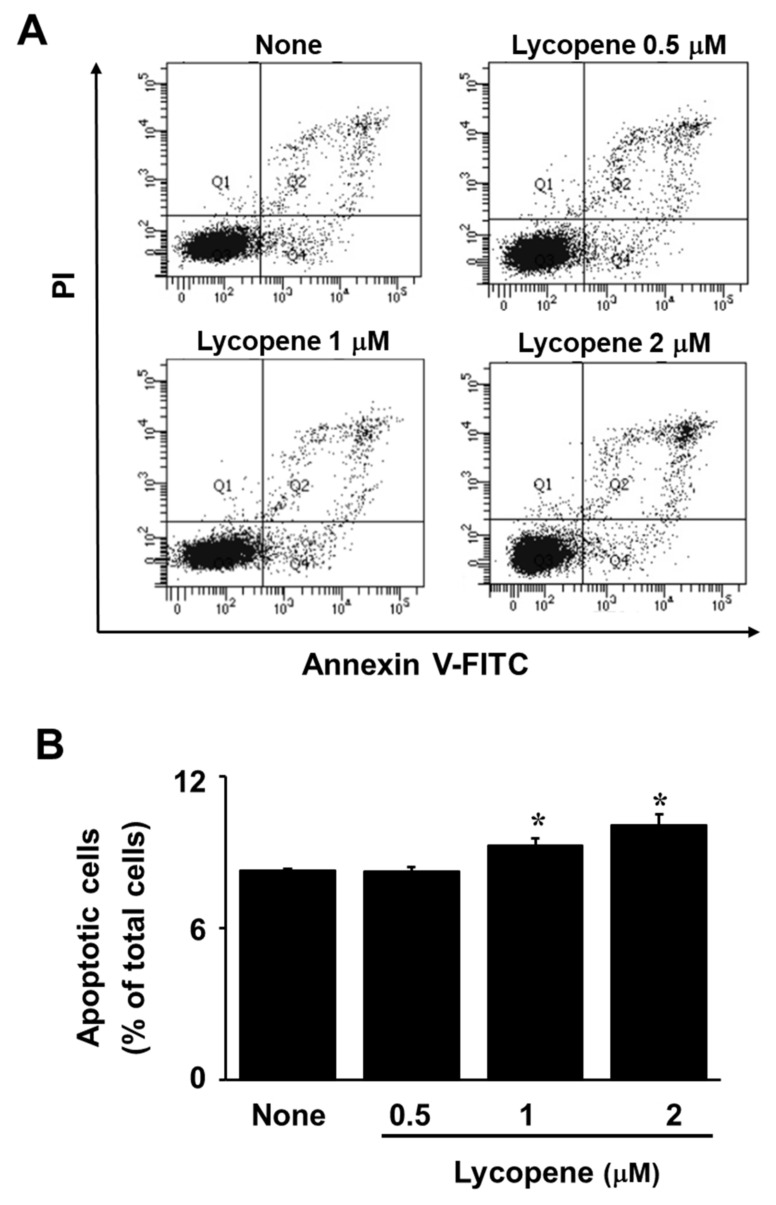
Lycopene induced apoptosis of AGS cells. (**A**) Flow cytometric analysis results of lycopen-induced apoptosis in AGS cells at different concentrations of lycopene are shown. (**B**) A bar graph of fluorescein isothiocyanate (FITC)–labeled annexin V/propidium iodide (PI) staining is shown. Apoptotic cells included the Annexin V^+^/PI^−^ cells (early apoptosis) and the Annexin V^+^/PI^+^ cells (late apoptosis). * *p* < 0.05 versus the cells treated with a vehicle tetrahydrofuran without lycopene (“none”).

**Figure 3:**
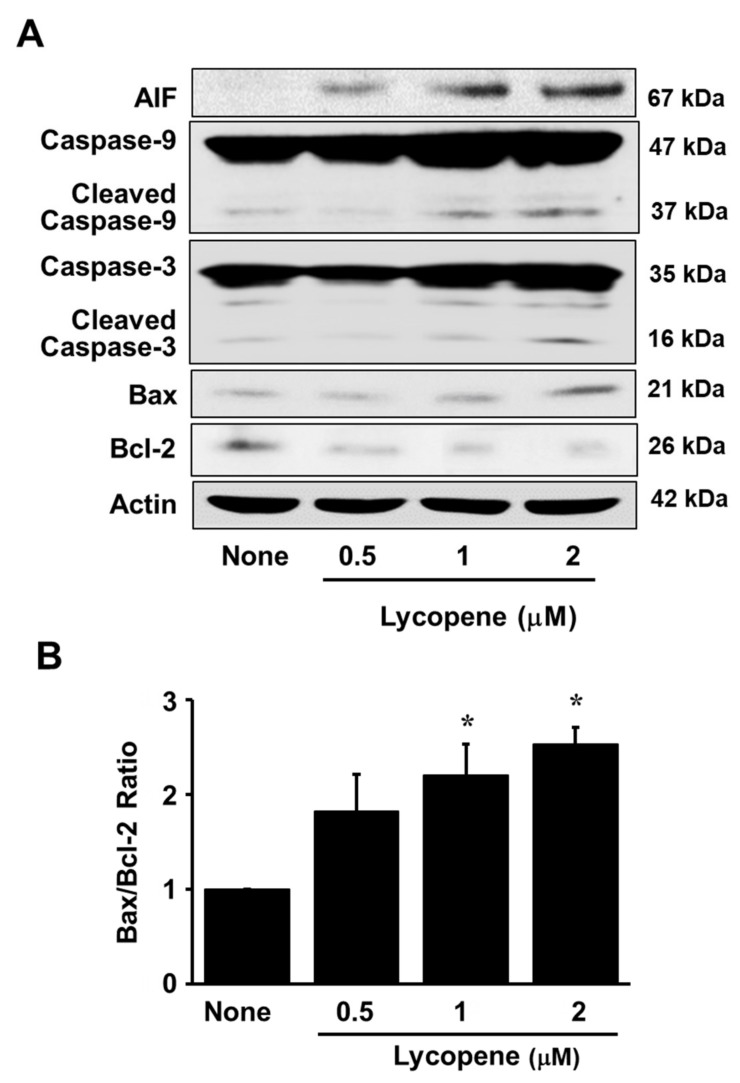
Lycopene increases apoptotic indices (apoptosis inducing factor (AIF), cleavage of caspase-3 and caspase-9, and Bax/Bcl-2 ratio). The cells were treated with the indicated concentrations of lycopene for 24 h. (**A**) The levels of AIF, total and cleaved caspase-3 and caspase-9, Bax, and Bcl-2 in whole-cell extracts were examined by Western blot analysis. Actin was served as a loading control. (**B**) The level of Bax was compared to that of Bcl-2 and expressed as the percentage density ratio. The ratio of Bax/Bcl-2 for untreated cells was set as 1. * *p* < 0.05 versus the cells treated with a vehicle tetrahydrofuran without lycopene (“none”).

The exposure of AGS cells to lycopene at increasing concentrations for 24 or 48 h reduced the number of viable cells in a dose-dependent manner (Figure 1A). Likewise, treatment with lycopene for 24 h increased DNA fragmentation in a dose-dependent manner (Figure 1B). For a further assessment of apoptosis induced by lycopene, we examined the exposure of phosphatidylserine on the cell surface by using Annexin V/PI double staining. Flow cytometry analysis revealed that the percentage of apoptotic cells with Annexin V-positive increased gradually in a dose-dependent manner (Figure 2A,B). In addition, levels of cleaved caspase-3 and caspase-9 were significantly elevated by treatment of lycopene (Figure 3A). Lycopene treatment also decreased Bcl-2 levels and increased Bax levels in AGS cells, leading to a marked increase in the Bax/Bcl-2 ratio (Figure 2A,B). Lastly, as shown in Figure 3A, the level of AIF was also increased by lycopene in a dose-dependent manner. These results indicate that lycopene inhibits cell proliferation and induces apoptosis.

### 3.2. Lycopene Reduces ROS Levels and Inhibits EGFR/Ras/ERK and p38 MAPK Signaling in AGS Cells

**Figure 4:**
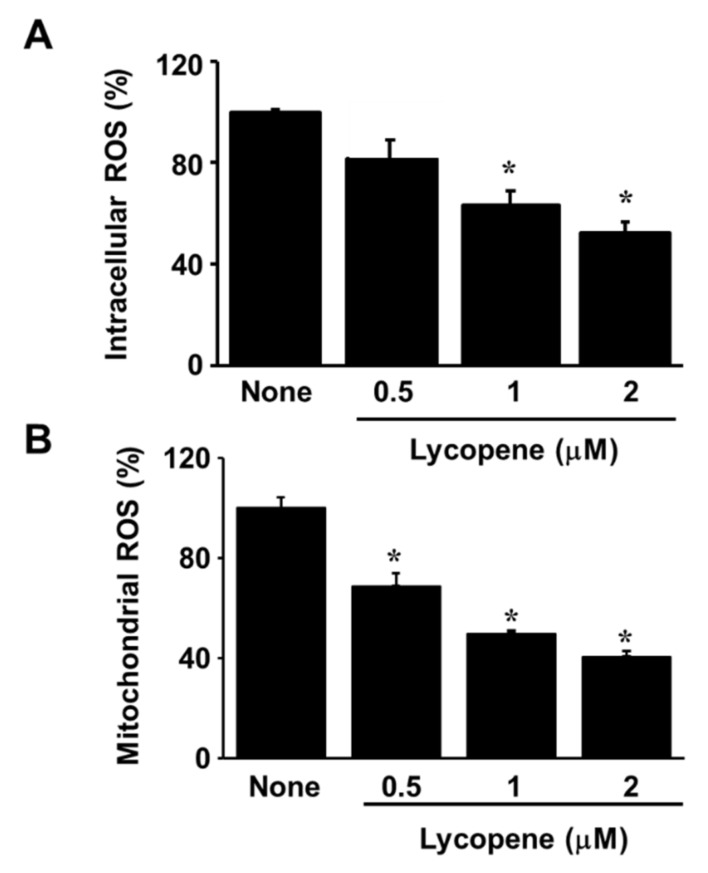
Lycopene reduces intracellular and mitochondrial reactive oxygen species (ROS) levels. The cells were treated with the indicated concentrations of lycopene for 1 h. The levels of intracellular (**A**) and mitochondrial (**B**) ROS were analyzed using the dichlorofluorescein and MitoSOX fluorescence, respectively. * *p* < 0.05 versus the cells treated with a vehicle tetrahydrofuran without lycopene (“none”).

**Figure 5:**
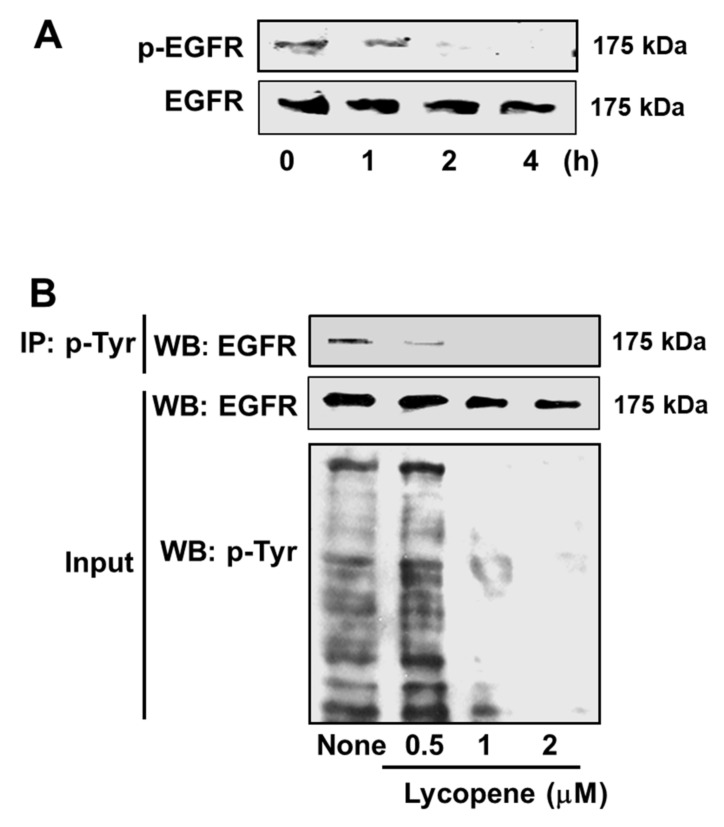
Lycopene inhibits epidermal growth factor receptor (EGFR) transactivation. (**A**) The cells were treated with 2 μM lycopene for the indicated time periods. The protein levels of p-EGFR and EGFR were examined by Western blot analysis. (**B**) The cells were treated with the indicated concentrations of lycopene for 2 h. Whole-cell extracts were subjected to immunoprecipitation (IP) with anti-phosphorylated tyrosine (p-Tyr) antibody, followed by subsequent Western blot analysis (WB) with anti-EGFR antibody (upper panel). The input of EGFR and p-Tyr was determined by Western blot analysis and served as control for the amounts of EGFR and p-Tyr (lower panel). The cells treated with a vehicle tetrahydrofuran without lycopene were expressed as “none”.

Lycopene reduced intracellular and mitochondrial ROS levels in AGS cells in a dose-dependent manner (Figure 4A,B). Western blot analysis for total EGFR, and for the phospho-specific form of EGFR (p-EGFR), was performed to identify whether the anti-proliferative and apoptotic effects of lycopene involve the EGFR signaling pathway. As shown in Figure 5A, the exposure of cells to 2 μM lycopene markedly decreased the activation of EGFR, as indicated by reduction in p-EGFR in a time-dependent manner. Total form of EGFR tended to be decreased by 2 μM lycopene treatment time-dependently. Figure 5B reveals a dose-dependent reduction in the amount of EGFR in the immunoprecipitated phosphotyrosine (p-Tyr)-containing protein (upper panel). The input of EGFR and phosphorylated tyrosine (p-Tyr), which served as control for the amounts of EGFR and p-Tyr (lower panel). Both total EGFR and p-Tyr reduced by lycopene in a concentration-dependent manner.

**Figure 6:**
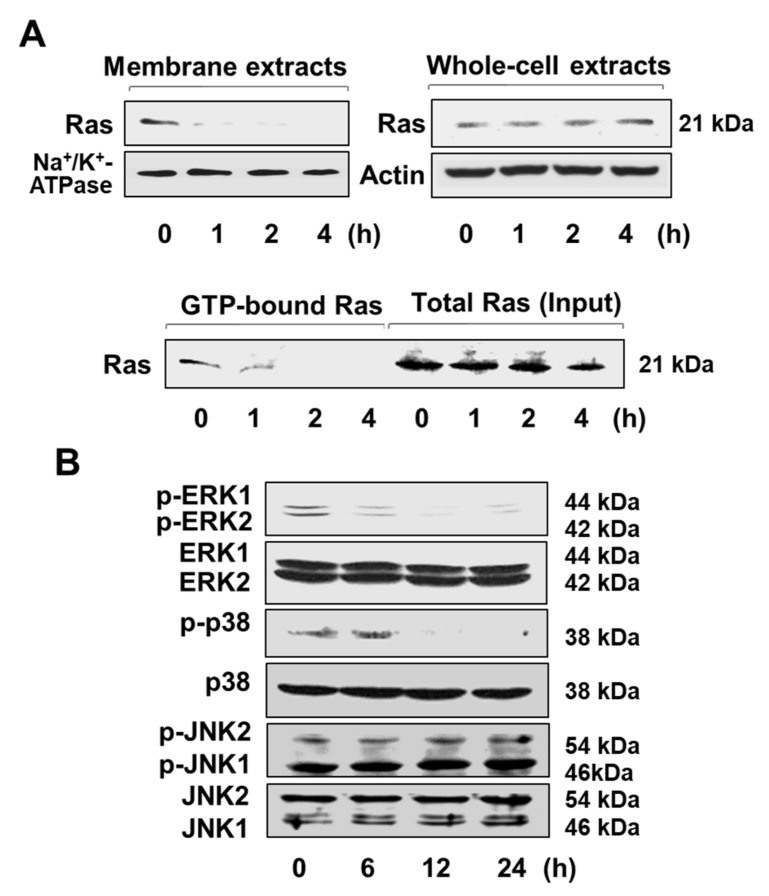
Lycopene inhibits the activation of Ras and mitogen-activated protein kinases (MAPKs). The cells were treated with 2 μM lycopene for the indicated time periods. (**A**) The levels of Ras in membrane extracts and whole-cell extracts were assessed by Western blot analysis using anti-Ras antibody (upper panel). Na^+^/K^+^-ATPase was used as a marker of membrane fraction. Actin served as a loading control. The activation of Ras was determined by affinity purification using glutathione S-transferase (GST) fusion protein containing the Ras binding domain (GST-RBD) followed by Western blot analysis. The Ras level in whole-cell extracts was considered as total Ras (input) (lower panel). (**B**) The phospho- and total forms of extracellular signal-regulated kinase (ERK) 1/2 (p44/p42), p38, and jun kinases (JNK) 2/1 (p54/p46) were assessed by Western blot analysis.

Ras is one of the key downstream mediators of EGFR. The mobilization of Ras to the plasma membrane is necessary for Ras activation. Western blot analysis shows that the amount of Ras in membrane extracts is significantly reduced by exposure of AGS cells to 2 μM lycopene whereas the level of Ras in the whole-cell extracts is unchanged (Figure 6A, upper panel). This finding indicates that lycopene may inhibit the translocation of Ras to the membrane. Moreover, lycopene decreased the amount of active GTP-bound Ras in a time-dependent manner, whereas the amount total Ras was not altered (Figure 6A, lower panel). 

The activation of Ras is known to stimulate signaling cascades via phosphorylation of MAPKs. As shown in Figure 6B, the treatment of AGS cells with 2 μM lycopene suppressed the activation of ERK1/ERK2 (p44/p42) and p38 MAPK in a time-dependent fashion, as evidenced by decreased levels of the phospho-specific forms of ERK1/2 and p38 MAPK. In contrast, the amounts of total JNK2/1 (p54/p46) and phosphorylated JNK2/1 were not altered. These results indicate that lycopene suppresses the ROS-mediated EGFR/Ras/ERK and p38 MAPK signaling pathways in AGS cells.

### 3.3. Lycopene Attenuates NF-κB p50/p50 Activity and COX-2 Gene Expression

**Figure 7:**
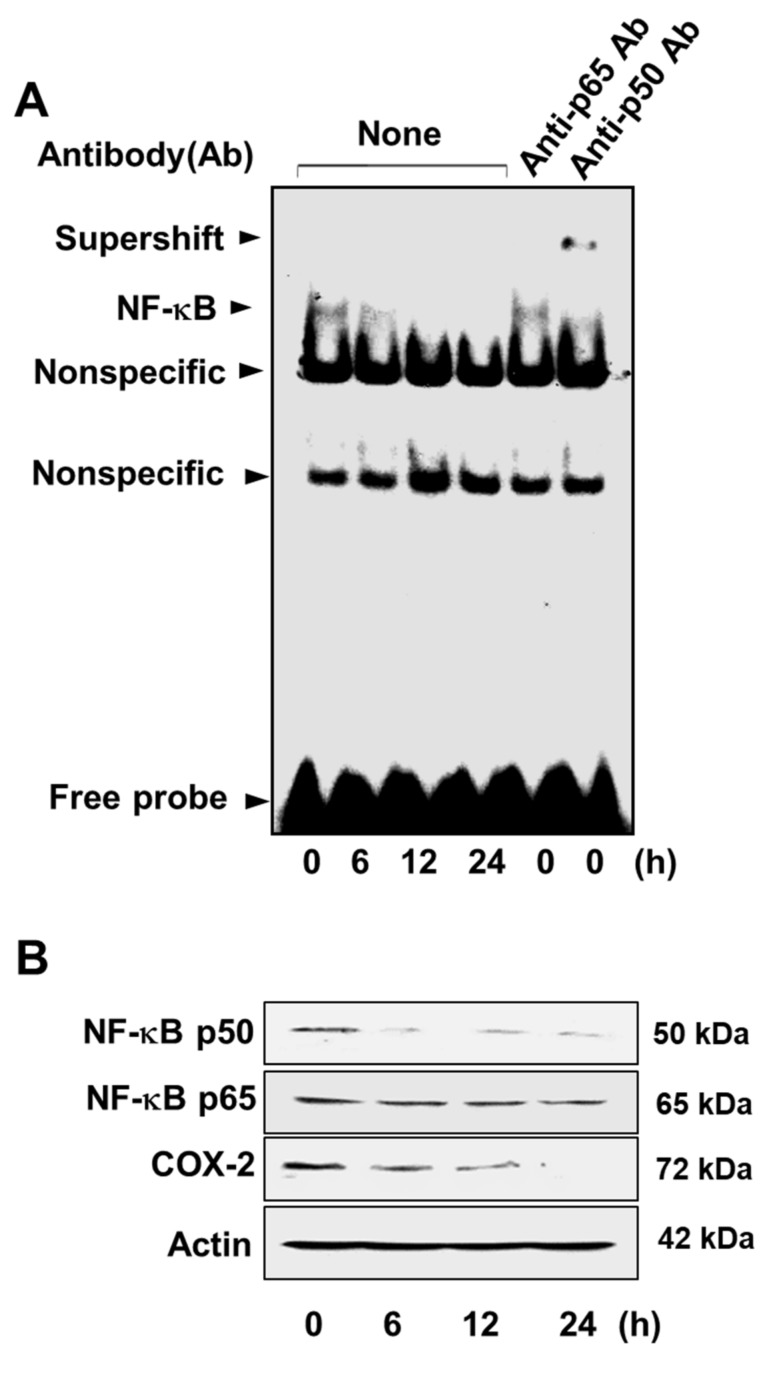
Lycopene decreases the DNA-binding activity of nuclear factor-kappa-light-chain-enhancer of activated B cells (NF-κB) and the levels of NF-κB subunit p50 and cyclooxygenase-2 (COX-2). The cells were treated with 2 μM lycopene for the indicated time periods. (**A**) DNA-binding activity of NF-κB in the nuclear extracts was determined by electrophoretic mobility shift assay. Antibodies (Ab) against the NF-κB subunits p50 and p65 were added to the nuclear extracts for the supershift assay (columns 5 and 6). “None” Ab means that no antibodies were added to the nuclear extracts (columns 1–4). (**B**) The levels of the NF-κB subunits p50 and p65 and of COX-2 in whole-cell extracts were examined by Western blot analysis. Actin was used as a loading control. “Nonspecific” means nonspecific binding.

Incubation of AGS cells with 2 μM lycopene over a 24 h period attenuated the DNA-binding capacity of NF-κB in a time-dependent manner (Figure 7A). The supershift with the anti-p50 antibody, but not with the anti-p65 antibody, indicates that the active form of NF-κB in the AGS cells is the p50/p50 homodimer (Figure 7A, columns 5 and 6, respectively). 

Western blots of cellular NF-κB p50 and p65 revealed that lycopene significantly decreased the level of NF-κB p50, whereas the level of NF-κB p65 was unaffected (Figure 7B). These results show that lycopene inhibits the activation of NF-κB by suppressing the formation of the NF-κB p50/p50 homodimer. In addition, lycopene induced decrease in the level of COX-2 (Figure 7B), which may be attributed to the decrease in active NF-κB, a key COX-2 transcriptional regulator. 

## 4. Discussion

In the present work, we found that lycopene inhibits cell proliferation and induces cell apoptosis by reducing the levels of ROS, and thereby inhibiting ROS-activated EGFR/Ras/ERK and p38 MAPK signaling pathways and suppressing NF-κB p50/p50-mediated COX-2 gene expression in gastric cancer AGS cells. 

Interestingly, Figure 1A showed that the cells treated with lycopene proliferate even though the proliferation rates of lycopene-treated cells were lower than those of the untreated cells. In addition, some cells treated with lycopene (2 μM) die in an apoptotic way (Figure 1B and Figure 2A,B). We also found that total ERK/JNK/p-38 levels were not changed by lycopene treatment (Figure 6B). Relatively large amounts of EGFR were present in lycopene-treated cells (Figure 5A,B). There results demonstrate that lycopene treatment did not make all cells die but reduced cell proliferation. Therefore, reducing ROS and decreasing p-Tyr-EGFR by lycopene treatment (Figure 4A,B and Figure 5A,B ) could not be the result from death of cells. Moreover, Figure 5B showed that lycopene treatment reduced p-Tyr and EGFR (Input, lower panel) as well as interaction of p-Tyr and EGFR (upper panel). It may or may not be related to reduction of NF-kB and COX-2 expression. 

In the present study, we used THF as a vehicle for lycopene. For lycopene treatment, vehicles such as THF, DMSO, or lipid micelles have been proposed, and most studies use THF as the solvent [38,39]. No differences were found between cells treated with THF (under 0.05%) and untreated cells in terms of cell number and viability [40]. 

The importance of lycopene inhibition of ROS-mediated activation of cell survival pathways in gastric cancer tissue is evident from previous in vivo investigations. Firstly, for rats having *N*-methyl-*N*′-nitro-*N*-nitrosoguanidine (MNNG)-induced gastric cancer, lycopene reduced oxidative injury to gastric tissues by decreasing the level of the oxidative parameter (malondialdehyde) and increased activities of antioxidant enzymes (SOD, catalase, and glutathione peroxidase) [41]. Administration of lycopene reduced stomach tumor size and incidence and modulated the redox status in the tumor and host tissues [42]

It is well-known that ROS act as essential signaling agents in the activation of oncogenic responses in cancer cells. One of the main targets activated by ROS in cancer is EGFR. Several observations have revealed that hydrogen peroxide, a non-radical ROS, induces phosphorylation at EGFR tyrosine residues in a ligand-independent manner [9,10]. It has also been demonstrated that treatment of AGS cells with hydrogen peroxide rapidly activates the EGFR/ERK MAPK signaling cascades [43]. Moreover, hydrogen peroxide oxidizes and inactivates several protein tyrosine phosphatases (PTPs) in a reversible manner [44]. PTPs catalyse the dephosphorylation of tyrosyl phosphorylated proteins. Thus, PTPs have been implicated in the dephosphorylation of the EGFR and the consequent suppression of EGFR signaling. It has been suggested that hydrogen peroxide oxidation of PTPs may be the underlying mechanism for ROS-induced phosphorylation of tyrosine residues in EGFR, an index of transactivation of EGFR. In the current work, we observed that lycopene suppresses the transactivation of EGFR in AGS cells, as indicated by the reduced level of phosphorylated EGFR. This finding is confirmed by the decrease in the amount of phosphorylated EGFR present in the phosphorylated tyrosine immunoprecipitate. Therefore, lycopene may inhibit EGFR activation by reducing ROS in AGS cells. 

Besides, lycopene reduced total form of EGFR both time and dose-dependent manner in the present study. Some evidences show that lycopene inhibits the expression of EGFR that is known to promote the survival, progression, and metastasis of prostate cancer [45,46]. Moreover, lycopene inhibited phosphorylation of EGFR in human cutaneous squamous cell carcinoma COLO16 cells [47]. These studies support the present results showing that lycopene inhibits both total and phosphor-specific forms of EGFR in AGS cells. Further studies should be performed to determine whether lycopene affects expression of EGFR at transcription, translation, and post-translation levels. 

Ras is an essential downstream effector of EGFR. For its activation, Ras must be translocated from the cytosol to the plasma membrane. [15,48] In the present study, lycopene was shown to inhibit the localization of Ras to the plasma membranes of AGS cells, as evidenced by the decreased amount of Ras observed in membrane extracts and by the reduction in GTP-bound Ras. These results are supported by an earlier investigation showing that lycopene inhibits the translocation of Ras from the cytosol to the membrane, thereby preventing Ras activation in prostate cancer cells [34]. Ras activation is required for the initiation of MAPKs cascades. Lycopene suppressed phosphorylation of MAPKs, resulting in apoptosis and cell cycle arrest in HGC-27 gastric cancer cells and LNCaP prostate cancer cells [34,49]. These findings are consistent with decrease in phospho-specific forms of ERK1/2 and p38 MAPK by lycopene treatment in gastric cancer AGS cells shown in the present study. 

MAPKs regulate the transcriptional activity of NF-κB. We found that lycopene inhibits the DNA-binding activity of NF-κB in AGS cells in the present study. Interestingly, the EMSA supershift assay demonstrated that lycopene predominantly suppresses the activity of the NF-κB p50/p50 homodimer. This conclusion is reinforced by the observed decrease in the level of NF-κB p50 and no change in the level of NF-κB p65. It has been reported that in the murine B-cell lymphoma cell line, the MEK/ERK pathway affects the DNA-binding activity of NF-κB p50/p50 by acting as an upstream signal [20]. Therefore, we inferred that the decrease in the activity of NF-κB p50/p50 by lycopene is mediated through the suppression of the ERK MAPK signaling pathway. 

One of the target genes under the control of NF-κB is the COX-2 gene. We previously demonstrated that inhibition of NF-κB activity by treatment of antisense oligodeoxynucleotide (ODN) for NF-κB subunit p50 or transfection of a mutated IκBα gene (MAD-3 mutant) inhibited cell growth of human gastric cancer AGS cells. In addition, treatment with COX-2 inhibitors such as indomethacin and NS-398 inhibited proliferation of human gastric cancer AGS cells. Furthermore, prostaglandin E2 prevented the inhibition of cell growth in cells treated with AS ODN for NF-κB subunit p50 or transfected with the mutated IκBα gene. This study demonstrated the link between Cox-2 activity and proliferation in gastric cancer AGS cells [50]. Additionally, it has been shown that NF-κB p50/p50 is predominantly associated with the COX-2 gene expression in LPS-stimulated macrophages [51].These findings indicated that NF-κB p50/p50 regulates COX-2 gene expression. In the present study, p50 expression was reduced by lycopene treatment (Figure 6B). The result suggests that more likely lycopene acts on p50 transcription or protein expression.”

COX-2 is closely correlated with cancer progression and is frequently overproduced in gastric cancer cells. A meta-analysis conducted with 27 published investigations demonstrated that the overexpression of the COX-2 gene is an independent prognostic factor for low survival rate in gastric cancer patients [52]. According to previous studies, overexpression of the COX-2 gene prevented apoptosis through upregulation of the Bcl-2 gene [53]. In addition, COX-2 inhibitors were found to suppress the tumor growth and induce apoptosis in a human gastric cancer xenograft model and in a gastric adenocarcinoma cell line, respectively [54,55]. Therefore, we speculate that the inhibition of COX-2 gene expression in gastric cancer cells induces apoptosis. In agreement with this proposal, our results indicate that lycopene significantly decreases cell viability and induces apoptosis in AGS cells, as evidenced by the observed increase in DNA fragmentation and in the Bax/Bcl-2 ratio. It is, therefore, suggested that the induction of apoptosis by treatment of lycopene is attributable to inhibition of NF-κB p50/p50-mediated COX-2 gene expression, which in turn leads to a reduction in Bcl-2 and increase in Bax. 

Notably, lycopene concentrations used in the current study are physiologically achievable in humans. The plasma concentration of lycopene is highly dependent on dietary consumption of lycopene-containing foods [56]. Although the concentrations of lycopene (0.5–2 μM) used in this study are slightly higher than 0.5 μM, the mean level of lycopene in human plasma determined for an ethnically diverse population [57], these values may be attainable through dietary supplementation of lycopene or consumption of lycopene-rich foods. A meta-analysis conducted on 12 clinical trials showed that plasma lycopene concentration increased from 0.053–0.69 μM to 0.27–1.428 μM through lycopene supplementation [58]. An increase of up to 2.1 μM was observed in one such trial [59]. This level is close to the concentration range (0.5–2 μM) tested in the current work. Furthermore, based on toxicity studies, no side effects were observed for oral intake of 3 g/kg/day of synthetic lycopene for 13 weeks [60]. The assumed no-observed-adverse-effect level (NOAEL) for a 70 kg man would be equivalent to 210 g/day [61]. These studies indicate that dietary intake of lycopene-rich foods or supplementation of lycopene may be a safe and effective treatment for gastric cancer.

In the present study, we have not used inhibitors or si/shRNA for EGFR to inhibit EGFR signaling in gastric cancer cells. Further studies should be performed using inhibitors or si/shRNA for EGFR to determine the direct role of EGFR on COX-2 expression. Moreover, in the present study, we propose that ROS are the main molecules for EGFR/Ras/ERK and p38 MAPK pathways and NF-κB-mediated COX-2 gene expression in AGS cells. To determine whether antioxidant effect of lycopene is related to inhibition of COX-2 expression, further study should be performed using ROS generators treated with lycopene for determination of EGFR/MAPK/NF-κB-mediated COX-2 gene expression in AGS cells. Since mitochondrial ROS were reduced by lycopene, it is necessary to determine the relation of mitochondrial dysfunction (reduced mitochondrial membrane potential) and apoptosis in lycopene-treated cells.

Taken together, the results demonstrate that lycopene ameliorates oxidative stress by reducing intracellular ROS levels. Specifically, our results show that the antioxidative action of lycopene elicits inhibition of cell proliferation and induction of apoptosis by suppressing ROS-mediated COX-2 expression. In addition, we found that lycopene reduces ROS and suppresses the transactivation of EGFR and activation of Ras, which modulates ERK/p38 MAPKs responsible for NF-κB-mediated COX-2 gene expression. The findings provide strong evidence that the consumption of lycopene–rich foods could prevent the development of gastric cancer associated with oxidative stress.

## References

[1] Jemal A., Bray F., Center M.M., Ferlay J., Ward E., Forman D. (2011). Global cancer statistics. CA Cancer J. Clin..

[2] Møller H., Heseltine E., Vainio H. (1995). Working group report on schistosomes, liver flukes and helicobacter pylori. Meeting held at IARC, Lyon, 7–14 June 1994. Int. J. Cancer.

[3] D’Elia L., Rossi G., Ippolito R., Cappuccio F.P., Strazzullo P. (2012). Habitual salt intake and risk of gastric cancer: A meta-analysis of prospective studies. Clin. Nutr..

[4] Ladeiras-Lopes R., Pereira A.K., Nogueira A., Pinheiro-Torres T., Pinto I., Santos-Pereira R., Lunet N. (2008). Smoking and gastric cancer: Systematic review and meta-analysis of cohort studies. Cancer Causes Control.

[5] Szatrowski T.P., Nathan C.F. (1991). Production of large amounts of hydrogen peroxide by human tumor cells. Cancer Res..

[6] Pelicano H., Carney D., Huang P. (2004). Ros stress in cancer cells and therapeutic implications. Drug Resist. Updat..

[7] Futagami S., Hiratsuka T., Shindo T., Horie A., Hamamoto T., Suzuki K., Kusunoki M., Miyake K., Gudis K., Crowe S.E. (2008). Expression of apurinic/apyrimidinic endonuclease-1 (ape-1) in h. Pylori-associated gastritis, gastric adenoma, and gastric cancer. Helicobacter.

[8] Ma Y., Zhang L., Rong S., Qu H., Zhang Y., Chang D., Pan H., Wang W. (2013). Relation between gastric cancer and protein oxidation, DNA damage, and lipid peroxidation. Oxid. Med. Cell Longev..

[9] Miyazaki Y., Hiraoka S., Tsutsui S., Kitamura S., Shinomura Y., Matsuzawa Y. (2001). Epidermal growth factor receptor mediates stress-induced expression of its ligands in rat gastric epithelial cells. Gastroenterology.

[10] Gamou S., Shimizu N. (1995). Hydrogen peroxide preferentially enhances the tyrosine phosphorylation of epidermal growth factor receptor. FEBS Lett..

[11] Seshacharyulu P., Ponnusamy M.P., Haridas D., Jain M., Ganti A.K., Batra S.K. (2012). Targeting the EGFR signaling pathway in cancer therapy. Expert Opin. Ther. Targets.

[12] Kim M.A., Lee H.S., Lee H.E., Jeon Y.K., Yang H.K., Kim W.H. (2008). EGFR in gastric carcinomas: Prognostic significance of protein overexpression and high gene copy number. Histopathology.

[13] Takehana T., Kunitomo K., Suzuki S., Kono K., Fujii H., Matsumoto Y., Ooi A. (2003). Expression of epidermal growth factor receptor in gastric carcinomas. Clin. Gastroenterol. Hepatol..

[14] Gao M., Liang X.J., Zhang Z.S., Ma W., Chang Z.W., Zhang M.Z. (2013). Relationship between expression of EGFR in gastric cancer tissue and clinicopathological features. Asian Pac. J. Trop. Med..

[15] Simanshu D.K., Nissley D.V., McCormick F. (2017). Ras proteins and their regulators in human disease. Cell.

[16] Jeong W.J., Ro E.J., Choi K.Y. (2018). Interaction between Wnt/β -catenin and RAS-ERK pathways and an anti-cancer strategy via degradations of beta-catenin and RAS by targeting the Wnt/β -catenin pathway. NPJ Precis. Oncol..

[17] Johnson G.L., Lapadat R. (2002). Mitogen-activated protein kinase pathways mediated by ERK, JNK, and p38 protein kinases. Science.

[18] Cargnello M., Roux P.P. (2011). Activation and function of the MAPKs and their substrates, the MAPK-activated protein kinases. Microbiol. Mol. Biol. Rev..

[19] Hsu T.C., Young M.R., Cmarik J., Colburn N.H. (2000). Activator protein 1 (ap-1)–and nuclear factor κb (nf-κb)–dependent transcriptional events in carcinogenesis. Free Radic. Biol. Med..

[20] Kurland J.F., Voehringer D.W., Meyn R.E. (2003). The MEK/ERK pathway acts upstream of NFκB1 (p50) homodimer activity and bcl-2 expression in a murine b-cell lymphoma cell line. MEK inhibition restores radiation-induced apoptosis. J. Biol. Chem..

[21] Kim H., Seo J.Y., Kim K.H. (1999). Effects of mannitol and dimethylthiourea on helicobacter pylori-induced il-8 production in gastric epithelial cells. Pharmacology.

[22] Sasaki N., Morisaki T., Hashizume K., Yao T., Tsuneyoshi M., Noshiro H., Nakamura K., Yamanaka T., Uchiyama A., Tanaka M. (2001). Nuclear factor-kappaB p65 (rela) transcription factor is constitutively activated in human gastric carcinoma tissue. Clin. Cancer Res..

[23] Kumar A., Takada Y., Boriek A.M., Aggarwal B.B. (2004). Nuclear factor-kappaB: Its role in health and disease. J. Mol. Med..

[24] Tanabe T., Tohnai N. (2002). Cyclooxygenase isozymes and their gene structures and expression. Prostaglandins Other Lipid Mediat..

[25] Thomas B., Berenbaum F., Humbert L., Bian H., Béréziat G., Crofford L., Olivier J.L. (2000). Critical role of C/EBPδ and C/EBPβ factors in the stimulation of the cyclooxygenase-2 gene transcription by interleukin-1β in articular chondrocytes. Eur. J. Biochem..

[26] Zha S., Yegnasubramanian V., Nelson W.G., Isaacs W.B., De Marzo A.M. (2004). Cyclooxygenases in cancer: Progress and perspective. Cancer Lett..

[27] Lim H.Y., Joo H.J., Choi J.H., Yi J.W., Yang M.S., Cho D.Y., Kim H.S., Nam D.K., Lee K.B., Kim H.C. (2000). Increased expression of cyclooxygenase-2 protein in human gastric carcinoma. Clin. Cancer Res..

[28] Ristimaki A., Honkanen N., Jankala H., Sipponen P., Harkonen M. (1997). Expression of cyclooxygenase-2 in human gastric carcinoma. Cancer Res..

[29] Sun W.H., Zhu F., Chen G.S., Su H., Luo C., Zhao Q.S., Zhang Y., Shao Y., Sun J., Zhou S.M. (2008). Blockade of cholecystokinin-2 receptor and cyclooxygenase-2 synergistically induces cell apoptosis, and inhibits the proliferation of human gastric cancer cells in vitro. Cancer Lett..

[30] Di Mascio P., Kaiser S., Sies H. (1989). Lycopene as the most efficient biological carotenoid singlet oxygen quencher. Arch. Biochem. Biophys..

[31] Kim J.H., Lee J., Choi I.J., Kim Y.I., Kwon O., Kim H., Kim J. (2018). Dietary carotenoids intake and the risk of gastric cancer: A case-control study in Korea. Nutrients.

[32] De Stefani E., Boffetta P., Brennan P., Deneo-Pellegrini H., Carzoglio J.C., Ronco A., Mendilaharsu M. (2000). Dietary carotenoids and risk of gastric cancer: A case-control study in Uruguay. Eur. J. Cancer Prev..

[33] Assar E.A., Vidalle M.C., Chopra M., Hafizi S. (2016). Lycopene acts through inhibition of ikappaB kinase to suppress NFκB1 signaling in human prostate and breast cancer cells. Tumour Biol..

[34] Palozza P., Colangelo M., Simone R., Catalano A., Boninsegna A., Lanza P., Monego G., Ranelletti F.O. (2010). Lycopene induces cell growth inhibition by altering mevalonate pathway and ras signaling in cancer cell lines. Carcinogenesis.

[35] Park Y.O., Hwang E.S., Moon T.W. (2005). The effect of lycopene on cell growth and oxidative DNA damage of Hep3B human hepatoma cells. Biofactors.

[36] Jang S.H., Lim J.W., Morio T., Kim H. (2012). Lycopene inhibits helicobacter pylori-induced ATM/ATR-dependent DNA damage response in gastric epithelial AGS cells. Free Radic. Biol. Med..

[37] Park B., Lim J.W., Kim H. (2018). Lycopene treatment inhibits activation of Jak1/Stat3 and Wnt/β -catenin signaling and attenuates hyperproliferation in gastric epithelial cells. Nutr. Res..

[38] Hantz H.L., Young L.F., Martin K.R. (2005). Physiologically attainable concentrations of lycopene induce mitochondrial apoptosis in LNCaP human prostate cancer cells. Exp. Biol. Med..

[39] Livny O., Kaplan I., Reifen R., Polak-Charcon S., Madar Z. (2002). Lycopene inhibits proliferation and enhances gap-junctional communication on KB-1 human oral tumor cells. J. Nutr..

[40] Burgess L.C., Rice E., Fischer T., Seekins J.R., Burgess T.P., Sticka S.J., Klatt K. (2008). Lycopene has limited effect on cell proliferation in only two of seven human cell lines (both cancerous and noncancerous) in an in vitro system with doses across the physiological range. Toxicol. In Vitro.

[41] Luo C., Wu X.G. (2011). Lycopene enhances antioxidant enzyme activities and immunity function in n-methyl-n’-nitro-n-nitrosoguanidine-enduced gastric cancer rats. Int. J. Mol. Sci..

[42] Velmurugan B., Nagini S. (2005). Combination chemoprevention of experimental gastric carcinogenesis by s-allylcysteine and lycopene: Modulatory effects on glutathione redox cycle antioxidants. J. Med. Food.

[43] Wang Q., Shen W., Tao G.Q., Sun J., Shi L.P. (2017). Study on the proliferation of human gastric cancer cells by activation of EGFR in H_2_O_2_. Eur. Rev. Med. Pharmacol. Sci..

[44] Denu J.M., Tanner K.G. (1998). Specific and reversible inactivation of protein tyrosine phosphatases by hydrogen peroxide: Evidence for a sulfenic acid intermediate and implications for redox regulation. Biochemistry.

[45] Hernes E., Fossa S.D., Berner A., Otnes B., Nesland J.M. (2004). Expression of the epidermal growth factor receptor family in prostate carcinoma before and during androgen independence. Br. J. Cancer.

[46] Rafi M.M., Kanakasabai S., Reyes M.D., Bright J.J. (2013). Lycopene modulates growth and survival associated genes in prostate cancer. J. Nutr. Biochem..

[47] Suyun B.I., Li L.I., Song X.U., Zhang M., Heng G.U., Zhou Z., Chen X. (2018). Regulatory effects of lycopene on the key signaling receptors in human cutaneous squamous cell carcinoma cell line COLO16. Chin. J. Dermatol..

[48] Willumsen B.M., Christensen A., Hubbert N.L., Papageorge A.G., Lowy D.R. (1984). The p21 ras c-terminus is required for transformation and membrane association. Nature.

[49] Zhang B., Gu Y. (2014). Low expression of ERK signaling pathway affecting proliferation, cell cycle arrest and apoptosis of human gastric HGC-27 cells line. Mol. Biol. Rep..

[50] Lim J.W., Kim H., Kim K.H. (2001). Nuclear factor-kappaB regulates cyclooxygenase-2 expression and cell proliferation in human gastric cancer cells. Lab. Invest..

[51] Kang Y.J., Wingerd B.A., Arakawa T., Smith W.L. (2006). Cyclooxygenase-2 gene transcription in a macrophage model of inflammation. J. Immunol..

[52] Song J., Su H., Zhou Y.Y., Guo L.L. (2014). Cyclooxygenase-2 expression is associated with poor overall survival of patients with gastric cancer: A meta-analysis. Dig. Dis. Sci..

[53] Tsujii M., DuBois R.N. (1995). Alterations in cellular adhesion and apoptosis in epithelial cells overexpressing prostaglandin endoperoxide synthase 2. Cell.

[54] Sawaoka H., Kawano S., Tsuji S., Tsujii M., Gunawan E.S., Takei Y., Nagano K., Hori M. (1998). Cyclooxygenase-2 inhibitors suppress the growth of gastric cancer xenografts via induction of apoptosis in nude mice. Am. J. Physiol..

[55] Li H.L., Chen D.D., Li X.H., Zhang H.W., Lu Y.Q., Ye C.L., Ren X.D. (2002). Changes of NF-kB, p53, Bcl-2 and caspase in apoptosis induced by jte-522 in human gastric adenocarcinoma cell line AGS cells: Role of reactive oxygen species. World J. Gastroenterol..

[56] Couillard C., Lemieux S., Vohl M.C., Couture P., Lamarche B. (2016). Carotenoids as biomarkers of fruit and vegetable intake in men and women. Br. J. Nutr..

[57] Hodge A.M., Simpson J.A., Fridman M., Rowley K., English D.R., Giles G.G., Su Q., O’Dea K. (2009). Evaluation of an ffq for assessment of antioxidant intake using plasma biomarkers in an ethnically diverse population. Public Health Nutr..

[58] Chen J., Song Y., Zhang L. (2013). Effect of lycopene supplementation on oxidative stress: An exploratory systematic review and meta-analysis of randomized controlled trials. J. Med. Food.

[59] Allen C.M., Schwartz S.J., Craft N.E., Giovannucci E.L., De Groff V.L., Clinton S.K. (2003). Changes in plasma and oral mucosal lycopene isomer concentrations in healthy adults consuming standard servings of processed tomato products. Nutr. Cancer.

[60] Mellert W., Deckardt K., Gembardt C., Schulte S., Van Ravenzwaay B., Slesinski R. (2002). Thirteen-week oral toxicity study of synthetic lycopene products in rats. Food Chem. Toxicol..

[61] Trumbo P.R. (2005). Are there adverse effects of lycopene exposure?. J. Nutr..

